# Angular pregnancy

**DOI:** 10.4274/tjod.42402

**Published:** 2016-12-15

**Authors:** İbrahim Alanbay, Mustafa Öztürk, Kazım Emre Karaşahin, Müfit Cemal Yenen

**Affiliations:** 1 Gülhane Military Medical Academy, Department of Gynecology and Obstetrics, Ankara, Turkey; 2 Etimesgut Sait Ertürk State Hospital, Clinic of Gynecology and Obstetrics, Ankara, Turkey

**Keywords:** Angular pregnancy, Cornual pregnancy, Ectopic pregnancy, antenatal hemorrhage, postpartum hemorrhage

## Abstract

Angular pregnancy is a rare condition in which the embryo is implanted in the lateral angle of the uterine cavity, medial to the uterotubal junction and round ligament, and causes life-threatening obstetric complications. It is important to differentiate this condition from interstitial and cornual pregnancy because they all result in emergency conditions. Although angular pregnancy can progress to term pregnancy, it may be associated with major obstetric complications such as uterine rupture, placental retention, postpartum hemorrhage, or may need further surgery and hysterectomy. This report describes a case of angular pregnancy from the 6^th^ gestational week and continued until delivery in the 32^nd^ gestational week. Sonographic findings, follow-up, and delivery concerns are described in this manuscript.

## INTRODUCTION

Angular pregnancy was first defined in 1898 by the American obstetrician Howard Kelly as implantation of the embryo just medial to the uterotubal junction, in the lateral angle of the uterine cavity^(1,2)^. Angular pregnancy is distinguished from interstitial pregnancy by embryoposition where lateral uterine enlargement of an angular pregnancy displaces the round ligament upward and outward, whereas interstitial tubal pregnancy is located lateral to the round ligament^(1)^. No absolute anatomic boundaries distinguish angular pregnancies from normal pregnancies, but the closer the location to the lateral angle of the uterus, the more it may cause visual asymmetry, symptoms, and adverse events when the pregnancy progresses^(3)^.

Angular pregnancy is potentially dangerous and may lead to complications during pregnancy and delivery, such as persistent pelvic pain and bleeding, spontaneous abortion, uterine rupture, retained placenta, placenta accreta, and severe bleeding leading to hysterectomy^(1,4,5)^. Diagnosis is difficult, many cases may actually go undiagnosed. To the best of our knowledge, no reports have delineated the entire natural course of angular pregnancy from early diagnosis to delivery. We aimed to discuss the possible outcomes of an angular pregnancy and highlight the problems encounered during follow-up.

## CASE REPORT

**Figure 1:**
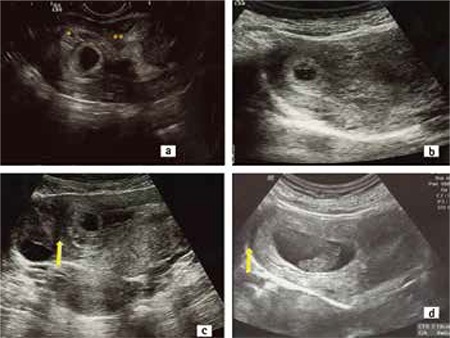
A) Transvaginal ultrasound view of the gestation sac at 6 weeks’ gestation. The sac is covered by the endometrium (*) and continuous with the intracavitary endometrial lining (**), B) Transabdominal ultrasound image of the angular pregnancy at 6 weeks’ gestation. The gestational sac is located in the right lateral side of the uterus. The myometrium surrounding the sac is thick and the uterus is asymmetric C) The gestastional sac growing towards the cavity at the 7th week, the ovary is seen lateral to the arrow. Note the thickness of the myometrium around the sac. D) Transabdominal ultrasound image of the angular pregnancy at 8+6 weeks’ gestation. The asymmetric uterine enlargement is distinct

**Figure 2:**
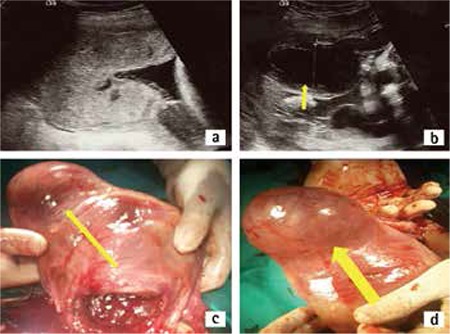
A) At 26 weeks’ gestational age, sonogram revealed a thickened and confined placenta at the right uterine angle B) Subchorionic hematoma at the placental edge C) Photograph of the angular pregnancy, anterior view of the uterus. The right cornual area protrudes as a sacculation. The uterus is distinctly asymetric D) The view of the posterior and right lateral side of the uterus. The area is discolored due to excessive vessel formation

A woman aged 34 years with a prior cesarean delivery, without symptoms, was admitted for a routine first antenatal examination in her 6^th^ gestational week. Endovaginal sonography showed a gestational sac located in the right lateral angle of the uterine cavity. The gestational sac was covered by endometrium of the medial aspect of the uterotubal junction, and the endometrial thickness was continuous with central endometrial lining (Figure 1A, B, C). We informed the patient about possible the diagnoses, natural courses, and complications. After discussing the risks, the patient requested to continue the pregnancy and close follow-up was decided. She presented with slight but disturbing abdominal pain and intermittent vaginal bleeding at 9 weeks. Sonography revealed a gestational sac in the right uterine angle, which was continuing to grow towards the cavity (Figure 1D). However, the uterine growth was asymmetrical. Vaginal spotting resolved after 2 weeks. The pregnancy’s development towards the uterine cavity continued, the base of the placenta was located in the right uterine angle (Figure 2A). The patient was admitted to the hospital at 27 weeks’ gestation because of vaginal bleeding and mild uterine contractions. Sonography revealed a 9x4 cm subcorionic hematoma, anterior and next to the edge of the placenta (Figure 2B). There was no placental abruption. Tocolysis was initiated and antenatal corticosteroid was given because the fetus was immature. The hematoma areas were stabilized aboutfor 5 weeks.

Intermittent vaginal bleeding continued. Fetal biometry continued to progress appropriate to the gestational week. A cesarean section was performed at 32 weeks of gestation because of uterine contractions and dilatation of the cervix. A 1650-g female fetus was delivered. The uterus was seen asymetrically enlarged, the right uterine angle region was bulging. Upon exteriorizing the uterus, a 9x9 cm sacculation was seen (Figure 2C, D). The vessels were excessive and the area was bluishly discolored due to the placental location. The placenta was delivered manually and with difficulty. This area was very thin and lacking myometrial tissue, as confirmed by intrauterine and extrauterine palpation. Due to the continuation of bleeding, 3 square compression sutures with absorbable 0 poliglecaprone were placed passing anterior to the posterior uterine wall where the bleeding was intense. Myometrial contraction was accomplished. Obliteration of this saccular area was confirmed through intrauterine digital examination. The surgery was completed without any further complications. Bleeding was not observed, and the patient was discharged after 72 h.

## DISCUSSION

Angular pregnancy is a rare and life-threatening obstetric complication in which the embryo is implanted in the lateral angle of the uterine cavity medial to the uterotubal junction and round ligament^(1)^. Contrary to interstistial pregnancy, which locate in the muscular layer of the origin of tuba uterina and surrounded by myometrial layer, in angular pregnancy the embryo locates in the lateral wall endometrial thickness of the uterus^(6,7)^. The surrounding endometrial tissue of embryo is continuous with the intracavitary endometrial line. A strict distinction between these three conditions is clinically important, because their findings, management, and outcomes are different^(7)^. Interstitial pregnancy may progress without symptoms until inevitable rupture occurs at 12-16 weeks^(6,7)^. Cornual pregnancy refers to a pregnancy in a rudimentary horn of a septate or bicornuate uterus^(6)^. In angular pregnancy, the embryo may abort or develop in the uterine cavity^(1)^. In contrast to interstitial pregnancy, angular pregnancy can progress to term^(1,4)^. If a patient presents at an advanced gestational age, the physician should suspect angular pregnancy if thickened placenta is located in an asymmetrically confined area of the uterine angle^(3)^. In the second and third trimester, the placenta may be seen limited to the uterine angle. Contrary to the normal placental growth pattern, the placenta of angular pregnancy must adopt a rigid uterine angle shape. In our opinion, the asymmetric appearance of the uterus, non-vertex fetal presentation, thickened placenta, placental adhesion anomalies, and muscular weakness of the area resulting from placental growth in the restricted, rather sharp edges of the uterine angle. This asymmetry can be seen and palpated in a thin patient in an abdominal examination. It is difficult to diagnose an angular pregnancy with certainty and to differentiate them from other abnormal implantations using ultrasound, because the main anatomic landmark (round ligament) is not visualized with this technique^(6)^. However, angular pregnancy can be accurately diagnosed with endovaginal sonography, especially during early gestational weeks. Alternatively, 3-D ultrasound and magnetic resonance exams can facilitate diagnosis, reduce the possibility of diagnosis failure, evaluate placenta implantation anomalies, and predict the risk of uterine rupture^(3,4,6,8)^. However, when magnetic resonance is not available, we believe that the most useful approach for an exact diagnosis is sequential ultrasound evaluations to determine whether the gestational growth is towards the uterine cavity. Angular pregnancies either terminate spontaneously or proceed to term. Even spontaneous termination might be complicated by improper separation of the placenta. A full-term delivery is likely if the gestational sac descends into the uterine cavity^(1,4)^. Jansen and Elliott^(1)^ reviewed 39 cases of suspected angular pregnancies and reported that 38.5% (10 of 26) had spontaneous or missed abortions, and 13.6% (3 of 22) had uterine ruptures. Recurrent bleeding can continue throughout pregnacy. The increased risk of preterm delivery, placental abruption, growth restriction, and postpartum endometritis is associated with angular pregnancy^(3,8)^. Abnormal fetal position can be seen, as our case was always in the breech presentation. Potential disadvantages of expectant management may include catastrophic complications such as uterine rupture. This management can be chosen by patient decision. It is necessary to counsel patients about the possible complications and close monitoring and frequent ultrasound examination should be conducted. What complicates the decision for expectant management is that there are no early sonographic signs to establish prognostic factors, although the risk of adverse outcomes can be expected to be higher when the degree of asymmetry of the protrusion at the angle is high, and the myometrium of the uterine angle is thin. It may be safer to terminate these pregnancies during the early stages. However, an inaccessible position of implantation may cause difficult curettage. Hysteroscopy and/or laparoscopy guided curettage, and treatment with methotrexate in early angular pregnancies are the preferred methods of treatment^(8)^. The site of angular pregnancy could cause uterine atony during delivery due to weakness or lack of myometrial tissue and inadequate contraction, and excessive vascular development. There may even be a need for hysterectomy if accompanied by a placental adhesion anomaly. In a case of suspected retained placenta, despite manual intervention, a coronal incision can be made into the myometrium overlying the placenta. In case of excessive bleeding due to atony, a few square sutures using long absorbable sutures from anterior to posterior through the uterus in order to obliterate the asymmetrical uterine sacculation can be peformed successfully as we did in our case.
